# Hyaluronic acid–green tea catechin conjugates as a potential therapeutic agent for rheumatoid arthritis†

**DOI:** 10.1039/d1ra01491a

**Published:** 2021-04-19

**Authors:** Fan Lee, Ki Hyun Bae, Shengyong Ng, Atsushi Yamashita, Motoichi Kurisawa

**Affiliations:** Institute of Bioengineering and Bioimaging 31 Biopolis Way, The Nanos Singapore 138669 Singapore mkurisawa@ibn.a-star.edu.sg +65-6478-9083 +65-6824-7139

## Abstract

Fibroblast-like synoviocytes are a key effector cell type involved in the pathogenesis of rheumatoid arthritis. The major green tea catechin, epigallocatechin-3-*O*-gallate (EGCG), has attracted significant interest for rheumatoid arthritis therapy because of its ability to suppress the proliferation and interleukin-6 secretion of synoviocytes. However, therapeutic efficacy of EGCG has been limited by a lack of target cell specificity. Herein we report hyaluronic acid–EGCG (HA–EGCG) conjugates as an anti-arthritic agent that is capable of targeting fibroblast-like synoviocytes *via* HA–CD44 interactions. These conjugates exhibited superior anti-proliferative and anti-inflammatory activities compared with EGCG under simulated physiological conditions. Near-infrared fluorescence imaging revealed preferential accumulation of the conjugates at inflamed joints in a collagen-induced arthritis rat model, and their anti-arthritic efficacy was investigated by measuring a change in the edema and histopathological scores. Our findings suggest the potential of HA–EGCG conjugates as an anti-arthritic agent for the treatment of rheumatoid arthritis.

## Abstract 2

Macromolecular HA–EGCG conjugates undergo targeted internalization by CD44-overexpressing fibroblast-like synoviocytes and subsequently cause H_2_O_2_-induced cell death and inhibition of IL-6 secretion, thereby suppressing the progression of arthritis.

## Graphical abstract

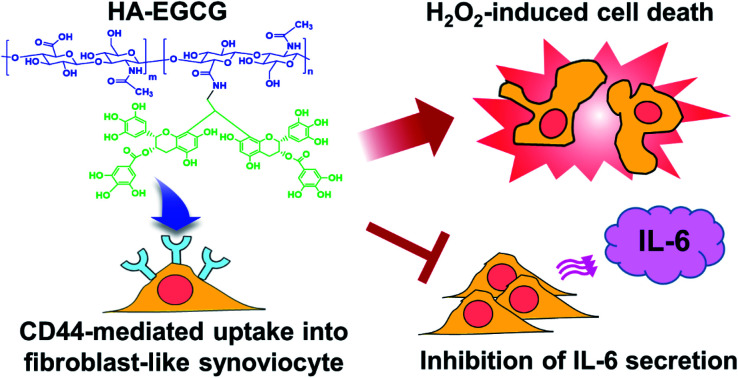

## Introduction

Rheumatoid arthritis (RA) is an autoimmune joint disease affecting approximately 0.5–1% of the world's adult population.^1^ Fibroblast-like synoviocytes (FLS), which constitute the synovial intimal lining of joints, play a central role in the pathogenesis of RA.^2^ During the progression of RA, FLS gain a hyper-proliferative phenotype and transform the synovial lining into an invasive hyperplastic tissue called pannus, which erodes articular cartilage and the underlying bone.^3,4^ Moreover, FLS facilitate joint inflammation and immune cell infiltration by secreting a repertoire of pro-inflammatory cytokines.^5^ Among them, interleukin-6 (IL-6) is of great concern because in addition to causing bone erosion by promoting osteoclast formation, it also induces cartilage degeneration and angiogenesis by stimulating the production of matrix metalloproteinases (MMPs) and pro-angiogenic factors, respectively.^6–8^ Therefore, suppression of FLS proliferation and IL-6 secretion represents a compelling therapeutic approach.

Epigallocatechin-3-*O*-gallate (EGCG) has received increasing attention for RA therapy because of its unique anti-proliferative and anti-inflammatory activities.^9^ Interestingly, EGCG induces caspase-dependent apoptosis of FLS and osteoclasts, while sparing osteoblastic cells essential for bone matrix synthesis.^10,11^ The apoptosis-inducing effect of EGCG appears to result primarily from its autoxidation, which generates cytotoxic levels of H_2_O_2_.^12,13^ Separately, EGCG also inhibits the production of IL-6, MMP-1 and MMP-3 from FLS.^14,15^ Furthermore, administration of EGCG reduces the incidence and severity of arthritis in animal models.^14,16,17^ Despite such promising properties, the use of EGCG for RA therapy has been hampered by a lack of FLS-targeting ability, which leads to non-specific systemic distribution and insufficient accumulation in inflamed joints.^18,19^

Hyaluronic acid (HA) is a non-sulfated polysaccharide mainly present in the extracellular matrix of connective tissues.^20^ Given its biocompatible and biodegradable properties, HA has been employed for various biomedical applications, including dermal augmentation, viscosupplementation, tissue engineering and drug targeting.^21–24^ Particularly, for RA therapy, there has been several attempts to use HA to deliver anti-arthritic agents into inflamed joints, because the HA receptor CD44 is over-expressed on FLS and activated macrophages in RA synovium.^25,26^ For instance, HA–methotrexate conjugates exhibited greater accumulation in the knees of collagen-induced arthritis (CIA) mice than those of non-diseased mice, and reduced the arthritis indices and IL-6 production more effectively than free methotrexate.^27^ Moreover, HA-coated solid lipid nanoparticles improved the anti-arthritic efficacy of encapsulated prednisolone by enhancing its accumulation in inflamed synovial tissues of CIA mice.^28^

**Fig. 1:**
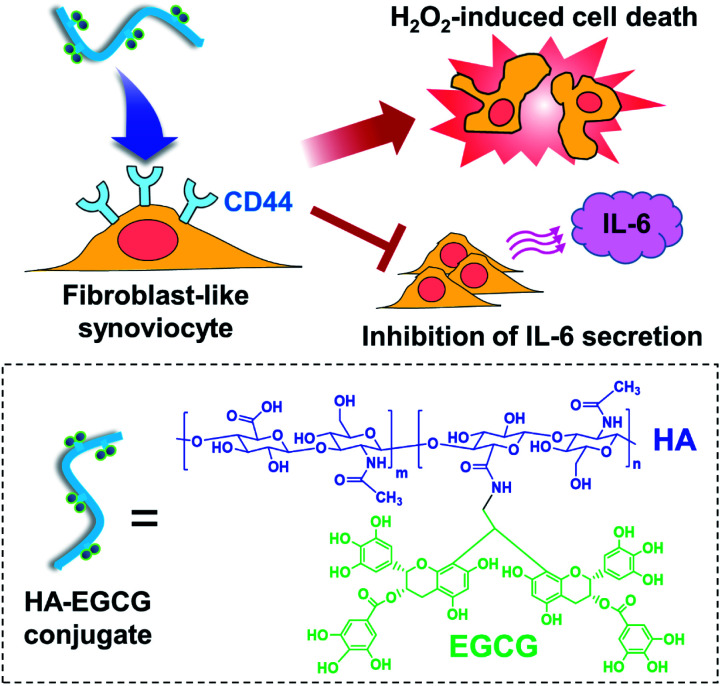
Proposed anti-arthritic effects of HA–EGCG conjugate on fibroblast-like synoviocytes. Following CD44-targeted cellular uptake, these conjugate is expected to exert anti-proliferative and anti-inflammatory activities by causing H_2_O_2_-induced cell death and inhibition of IL-6 secretion, respectively.

We have previously developed a method for synthesizing amine-functionalized EGCG dimers and their conjugation to carboxylic acid groups of HA backbone using carbodiimide-based coupling reactions.^29^ The resulting HA–EGCG conjugates were more effective than EGCG in scavenging superoxide (O_2_˙^−^) and hydroxyl radical (˙OH), suggesting their potential to attenuate free radical-induced cartilage damage in inflamed joints.^30^ Accumulating evidence suggest that conjugation of macromolecules with anti-arthritic drugs improves drug targeting to inflamed tissues in animal models of RA, *via* the extravasation through leaky angiogenic vasculature and subsequent inflammatory cell-mediated sequestration (ELVIS) effect.^31,32^ In this study, we investigated the applicability of HA–EGCG conjugates for RA therapy. We hypothesized that, in addition to preferential accumulation in inflamed synovium *via* the ELVIS effect, macromolecular HA–EGCG conjugates would also undergo targeted internalization by CD44-overexpressing FLS *via* HA–CD44 interactions and subsequently cause H_2_O_2_-induced cell death and inhibition of IL-6 secretion (Fig. 1), thereby suppressing the progression of arthritis. To this end, we examined the H_2_O_2_-generating property of HA–EGCG conjugates and their anti-proliferative and anti-inflammatory effects on FLS under simulated physiological conditions. We further assessed the *in vivo* distribution and anti-arthritic efficacy of HA–EGCG in a CIA rat model.

## Results and discussion

### CD44-targeted cellular uptake

**Fig. 2:**
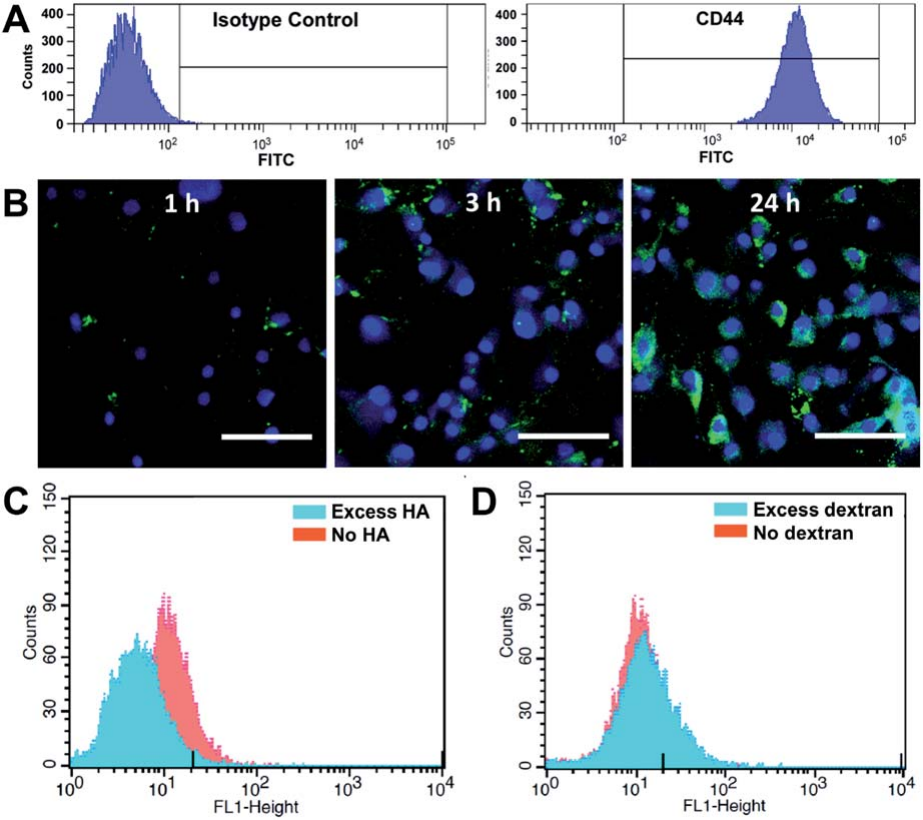
CD44-targeted cellular uptake. (A) Flow cytometric detection of CD44 in FLS after labeling with isotype control antibody (left panel) or anti-CD44 antibody (right panel). (B) Intracellular localization of HA–EGCG–AF conjugates (green) in FLS after 1 h, 3 h and 24 h incubation. The nuclei were labelled with 4,6-diamidino-2-phenylindole (blue). Scale bar = 50 μm. Effect of pretreatment of (C) HA and (D) dextran on the cellular uptake of HA–EGCG–AF conjugates.

We first investigated whether HA–EGCG conjugates could internalize into FLS in a CD44-specific manner. Flow cytometry confirmed highly elevated CD44 expression on FLS (Fig. 2A), consistent with the literature.^25,26^ To visualize intracellular localization, HA–EGCG conjugates were tagged with 5-aminofluorescein (AF) dyes. The resultant HA–EGCG–AF conjugates showed fluorescence emission spectrum peaked at 515 nm, confirming successful labeling of AF dyes (Fig. S1A†). As shown in Fig. 2B, cellular uptake of HA–EGCG–AF conjugates occurred as early as 1 h after incubation with FLS. At 3 h, the conjugates were predominantly sequestered in endo-lysosomal vesicles, as evident from the punctate fluorescence pattern.^33^ On the other hand, cells treated for 24 h showed a diffuse rather than a punctate fluorescence pattern, suggesting cytosolic localization of the conjugates. Cellular uptake of HA–EGCG–AF conjugates was decreased by pretreatment with excess HA (CD44 blocker), but not with excess dextran (non-CD44 binding polysaccharide) (Fig. 2C and D). These results demonstrated that the intracellular entry of HA–EGCG–AF conjugates was mainly through CD44-mediated endocytosis.

### Albumin binding and H_2_O_2_-producing properties

Interaction of EGCG with plasma components poses significant challenges for its *in vivo* application.^34^ Among them, albumin is of specific interest because it is the most abundant protein in blood, constituting about 50–60% of total plasma content.^35^ Albumin has been shown to bind to EGCG and inhibit its autoxidation process.^36,37^ Moreover, the addition of albumin diminished the cytotoxicity of EGCG by abrogating the intracellular production of reactive oxygen species.^38^ These studies suggest that binding of albumin would occur when systemically administering EGCG, resulting in potential loss of its therapeutic effects before reaching diseased tissues.

**Fig. 3:**
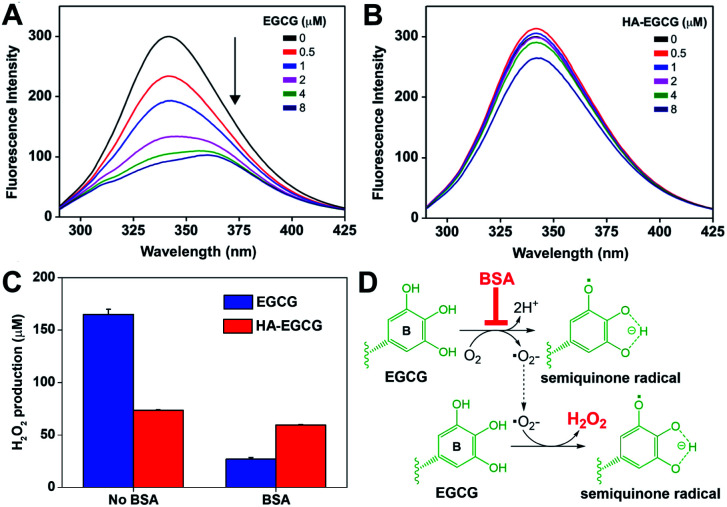
Albumin binding and H_2_O_2_-producing properties. Quenching of BSA fluorescence with increasing concentrations of (A) EGCG or (B) HA–EGCG conjugates. (C) H_2_O_2_ production by EGCG and HA–EGCG conjugates after 90 min incubation in medium with or without 2% BSA (*n* = 2, mean ± SD). (D) Possible mechanism for the inhibitory effect of BSA on H_2_O_2_ production of EGCG.

In this perspective, we performed fluorescence spectroscopy to investigate the interactions between albumin and HA–EGCG conjugates. As shown in Fig. 3A, BSA exhibited fluorescence emission at 342 nm, originating from two intrinsically fluorescent tryptophan residues.^39^ Increasing EGCG concentrations gradually quenched BSA fluorescence with a red shift of the emission peak wavelength to 359 nm, indicating binding between EGCG and BSA.^40^ In contrast, BSA fluorescence was marginally quenched by HA–EGCG conjugates (Fig. 3B) and had little influence on the fluorescence intensity ratio (*F*_0_/*F*) in the Stern–Volmer plot (Fig. S2†), suggesting that HA conjugation effectively prevented EGCG–BSA interactions. As both HA and BSA are negatively charged at physiological pH,^41^ their electrostatic repulsion may have played a role in the decreased binding of BSA to HA–EGCG conjugates.

Oxidative stability of EGCG has been considered an important issue when applying EGCG-based formulations for biomedical applications. For example, EGCG readily undergoes autoxidation at physiological pH and temperature, resulting in the formation of H_2_O_2_ as a byproduct.^29^ We evaluated the oxidative stability of HA–EGCG conjugates by measuring the rate of H_2_O_2_ production. Interestingly, HA–EGCG conjugates produced H_2_O_2_ more slowly than EGCG in phosphate-buffered saline (PBS) at 37 °C (Fig. S3†). Considering the inherent antioxidant activity of HA,^42,43^ it is plausible that conjugation of HA might confer protection of EGCG moieties from autoxidation. By contrast, such an effect was not observed from a physical mixture containing equivalent amounts of EGCG and unmodified HA. In this study, HA–EGCG conjugates were dissolved in deionized water (pH 5.8) at 25 °C and then diluted with cell culture medium immediately before use. Only negligible amounts of H_2_O_2_ were formed even after 6 h storage of HA–EGCG conjugate solution in deionized water (pH 5.8) at 25 °C (Fig. S4†), suggesting that its integrity was well preserved under the storage condition.

Next, we examined the effect of BSA on the H_2_O_2_-producing property of HA–EGCG conjugates in cell culture conditions. Consistently with the earlier finding in PBS, HA–EGCG conjugates produced smaller amounts of H_2_O_2_ (74 μM) than EGCG (165 μM) when incubated in RPMI medium for 90 min (Fig. 3C). However, this trend was reversed in BSA-supplemented medium, where H_2_O_2_ production of EGCG dropped dramatically to 27 μM, whereas H_2_O_2_ production of HA–EGCG conjugates only slightly decreased to 59 μM. This revealed that these conjugates were more effective in generating H_2_O_2_ than EGCG in the presence of BSA. The inhibitory effect of BSA on H_2_O_2_ generation was likely attributed to its binding to EGCG moieties. As depicted in Fig. 3D, EGCG undergoes autoxidation in the presence of oxygen, leading to the formation of semiquinone radical at the B-ring and H_2_O_2_.^44^ The binding of BSA mainly occurs at the B-ring of EGCG and consequently prevents its conversion to semiquinone radical during the autoxidation process.^36,45^ Hence, the superior H_2_O_2_-producing property of HA–EGCG conjugates is thought to result from their reduced interactions with BSA.

### *In vitro* anti-proliferative effect

**Fig. 4:**
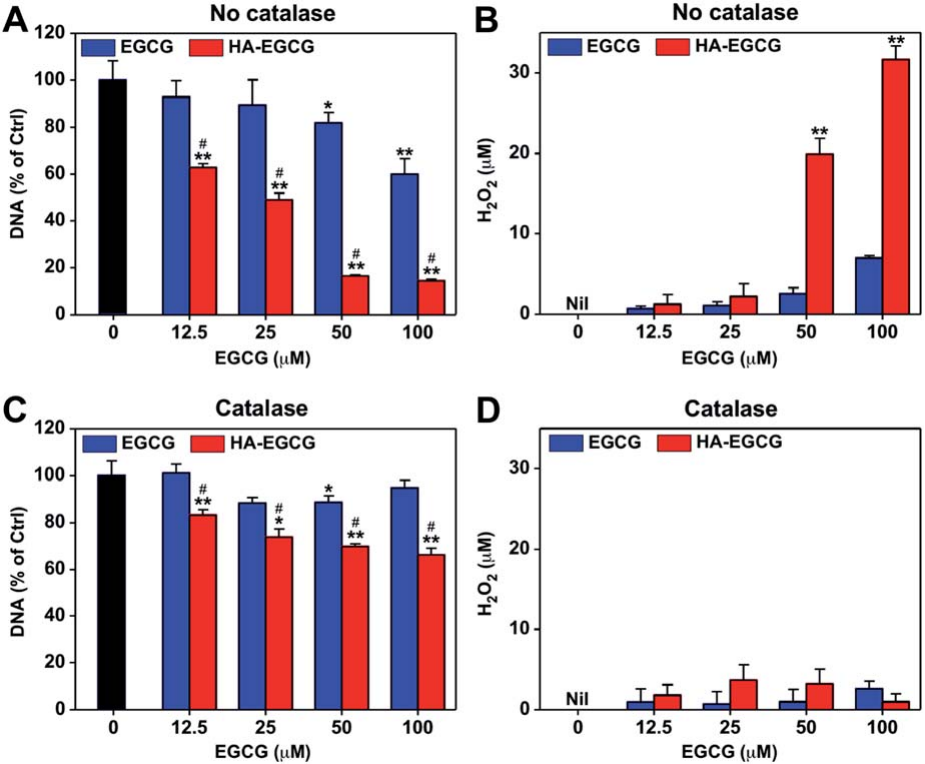
*In vitro* anti-proliferative effect. (A) DNA amount and (B) H_2_O_2_ production after 72 h treatment of TNFα-stimulated FLS with EGCG or HA–EGCG conjugates in BSA-supplemented complete growth medium. (C) DNA amount and (D) H_2_O_2_ production measured under the same condition, except for addition of catalase. Mean ± SD (*n* = 5). *P < 0.05, **P < 0.01 *versus* untreated control. ^#^*P* < 0.01 *versus* EGCG.

Spurred by the above finding, we sought to evaluate the anti-proliferative effect of HA–EGCG conjugates in BSA-supplemented medium. RA patients are known to have lower levels of serum albumin (24–38 g L^−1^) relative to healthy individuals (39–45 g L^−1^), due to increased albumin consumption at inflamed tissues.^46,47^ In this study, we supplemented the complete growth medium (10% FBS RPMI medium containing ∼4.6 g L^−1^ of BSA) with an additional 2% BSA to more closely mimic the physiological concentration of serum albumin in RA patients, and further added TNFα to simulate the pro-inflammatory RA environment.^48^ We treated FLS with either EGCG or HA–EGCG conjugates in the simulated physiological environment for 3 days and examined their proliferation by measuring DNA amounts. As presented in Fig. 4A, EGCG treatment decreased DNA amounts in a dose-dependent manner, in accordance with the previous studies describing the FLS-killing activity of EGCG.^10,11^ Notably, HA–EGCG conjugates exerted remarkably greater cytotoxicity than EGCG at equivalent doses. Markedly elevated levels of H_2_O_2_ were detected in the spent media of HA–EGCG-treated cells compared to EGCG-treated cells (Fig. 4B), suggesting the involvement of H_2_O_2_ in EGCG-induced cell death.

To ascertain the role of H_2_O_2_ in the observed anti-proliferative effect of EGCG, the cell growth assay was performed under the same conditions, except for addition of catalase to scavenge H_2_O_2_.^10^ The addition of catalase annulled the dose-dependent cytotoxic effect of EGCG, restoring DNA amounts to over 85% of the untreated control at all tested doses (Fig. 4C). This demonstrated that H_2_O_2_-producing property of EGCG was primarily responsible for its cytotoxicity, as previously reported.^12,13^ However, catalase treatment only partially reversed the growth-inhibitory effect of HA–EGCG conjugates, although H_2_O_2_ was scavenged to a level similar to that of EGCG (Fig. 4D). Thus, other mechanisms besides H_2_O_2_ production may have contributed to the enhanced anti-proliferative effect of HA–EGCG conjugates. A previous study has shown that EGCG triggers caspase-mediated apoptosis of FLS by downregulating the anti-apoptotic protein Mcl-1.^11^ Several other studies have implicated the involvement of intracellular signaling pathways in the growth-inhibitory effect of EGCG.^49,50^ In this context, the H_2_O_2_-independent anti-proliferative effect of HA–EGCG conjugates might be related to the inhibition of anti-apoptotic proteins and activation of apoptotic signaling pathways.

### *In vitro* anti-inflammatory effect

**Fig. 5:**
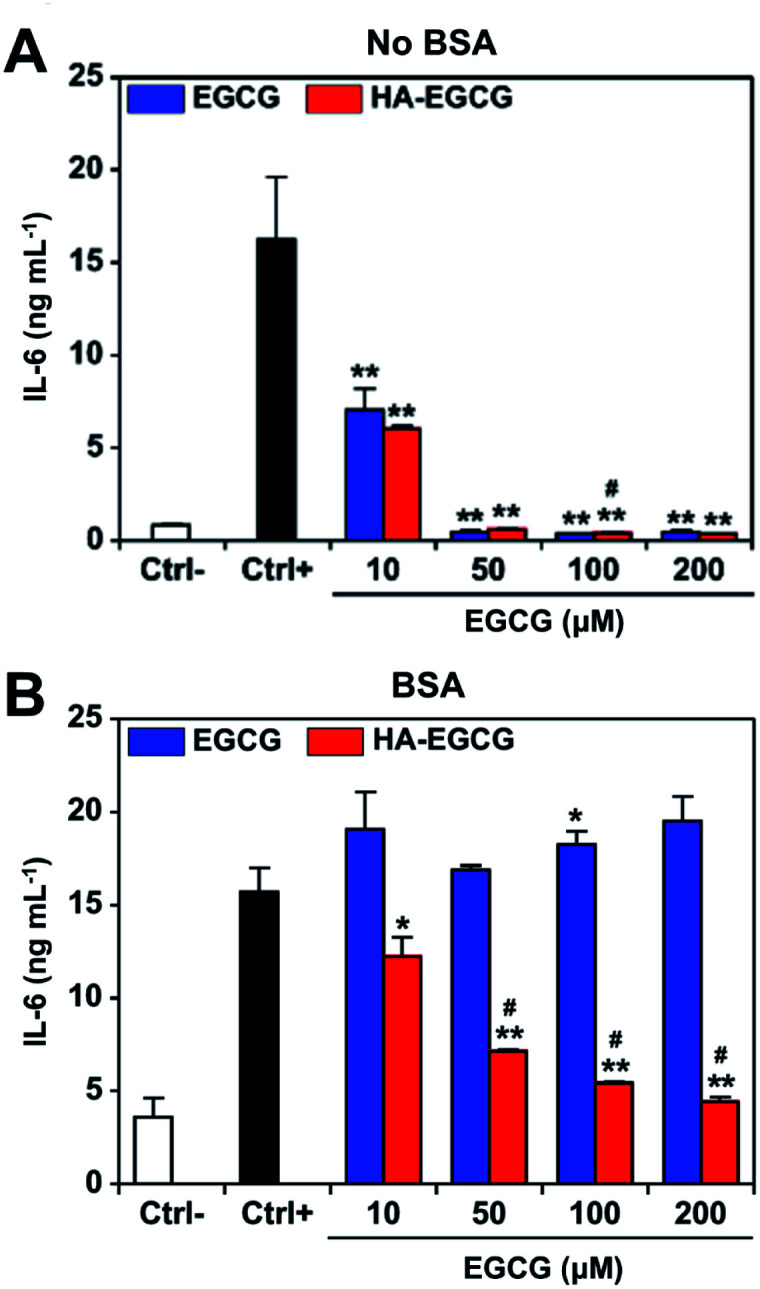
*In vitro* anti-inflammatory effect. IL-6 production after 24 h treatment of TNFα-stimulated FLS with EGCG or HA–EGCG conjugates in (A) RPMI medium and (B) BSA-supplemented medium (*n* = 2, mean ± SD). To exclude the effect of cytotoxicity caused by H_2_O_2_, catalase (100 units per mL) was added into both media at the beginning of cultivation. Ctrl−: negative control (unstimulated FLS), Ctrl+: positive control (TNFα-stimulated FLS). *P < 0.05, **P < 0.01 *versus* the positive control. ^#^*P* < 0.05 *versus* EGCG.

We first investigated the effect of HA–EGCG conjugates on IL-6 production of FLS in RPMI medium without BSA. To exclude the effect of H_2_O_2_-induced cytotoxicity, catalase was added into the medium at the beginning of cultivation. FLS secreted high levels of IL-6 upon TNFα stimulation (Fig. 5A), in agreement with the literature.^51^ Treatment with 10 μM EGCG or HA–EGCG conjugates suppressed the production of IL-6 by 57% and 63%, respectively. At concentrations of 50 μM and above, both EGCG and HA–EGCG conjugates decreased the level of IL-6 down to that observed in the unstimulated cells. The viability of FLS remained higher than 80% for all the conditions (Fig. S5†), suggesting that the observed inhibition of IL-6 production was not caused by cytotoxicity induced by H_2_O_2_ generated from EGCG moieties, but rather the regulation of intracellular IL-6 signaling by EGCG moieties. Indeed, qPCR analysis showed a notable decline in IL-6 mRNA levels in FLS treated with HA–EGCG conjugates, demonstrating that the reduced IL-6 production was due to the downregulation of IL-6 gene expression (Fig. S6†).

In BSA-supplemented medium containing catalase, EGCG failed to block IL-6 production at all tested doses, suggesting that anti-inflammatory effect of EGCG was almost completely eliminated due to its binding to BSA (Fig. 5B). In contrast, HA–EGCG conjugates significantly inhibited IL-6 production, albeit to a smaller extent compared to when BSA was absent. The enhanced anti-inflammatory effect of HA–EGCG conjugates was not attributable to direct anti-inflammatory activity by HA, since equivalent amounts of HA had a negligible impact on IL-6 production (Fig. S7†). Considering that HA–EGCG conjugates exhibited superior resistance to BSA adsorption over EGCG, it is reasonable to speculate that HA conjugation effectively protected EGCG moieties from BSA-mediated inactivation, thereby allowing them to exert anti-inflammatory effects.

### *In vivo* distribution study

**Fig. 6:**
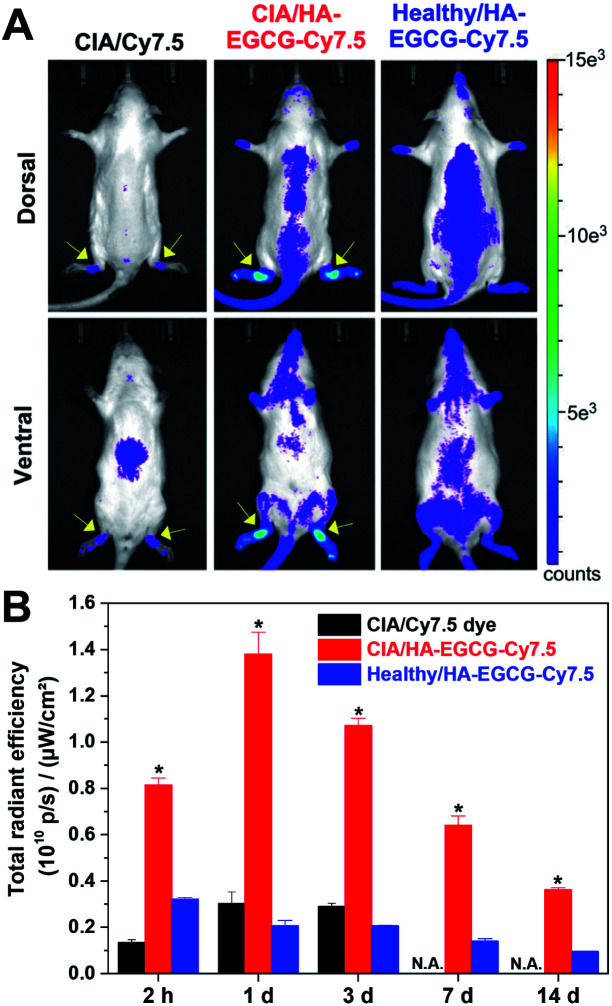
Near-infrared fluorescence imaging. (A) Dorsal and ventral images of CIA and healthy rats at 2 h post-injection of Cy7.5 dye or HA–EGCG–Cy7.5 conjugates. The yellow arrows indicate the location of ankles in CIA rats. (B) Fluorescence intensity in the hind paws over 2 weeks. Values for day 7 and day 14 of the CIA rat injected with Cy7.5 dye are not available (N.A.) because the rat was euthanized due to ulcer formation at the immunization site. *P < 0.05 *versus* the other groups.

To visualize their *in vivo* distribution, HA–EGCG conjugates were tagged with the near-infrared fluorophore Cy7.5. The resulting HA–EGCG–Cy7.5 conjugates displayed near-infrared fluorescence with emission maximum at 808 nm (Fig. S1B†). HA–EGCG–Cy7.5 conjugates or free Cy7.5 dye were intravenously injected to CIA rats on day 15 post-immunization when their ankle joints were visibly swollen. At 2 h post-injection of Cy7.5 dye, fluorescence was mainly detected in the abdominal region corresponding to the liver, whereas little fluorescence was seen at the hind paws (Fig. 6A). Injection of HA–EGCG–Cy7.5 conjugates led to more intense fluorescence signal at the hind paws of CIA rats compared to healthy rats, indicating preferential accumulation of these conjugates in the inflamed joints. Accumulation of HA–EGCG–Cy7.5 conjugates in the inflamed joints of CIA rats peaked at day 1, with about 6.7-fold greater accumulation than healthy rats, and gradually decreased over a period of 2 weeks (Fig. 6B), demonstrating their arthritis-targeting ability. The arthritis-targeting ability of HA–EGCG conjugates could be due to their enhanced permeation through leaky angiogenic vessels *via* the ELVIS effect and selective uptake into CD44-overexpressing FLS and activated macrophages in the inflamed synovium.^25,26^

### *In vivo* anti-arthritic efficacy

Lastly, we investigated the anti-arthritic efficacy of EGCG and HA–EGCG conjugates in CIA rats. The dose of EGCG (100 mg kg^−1^) and route of administration (intraperitoneal injection) were selected based on a previous study reporting therapeutic effects of EGCG in a rat RA model.^14^ Unexpectedly, we found that five of the rats died within 24 h of the first injection and the remaining two rats became moribund and had to be euthanized within a few days. This finding indicated that the dose of EGCG was lethal, although the previous study did not report any signs of toxicity.^14^ The reason for these conflicting results is unknown. A lower dose of EGCG (10 mg kg^−1^) has been reported non-toxic yet ineffective in reducing the joint swelling and arthritic score in a rat RA model.^52^

**Fig. 7:**
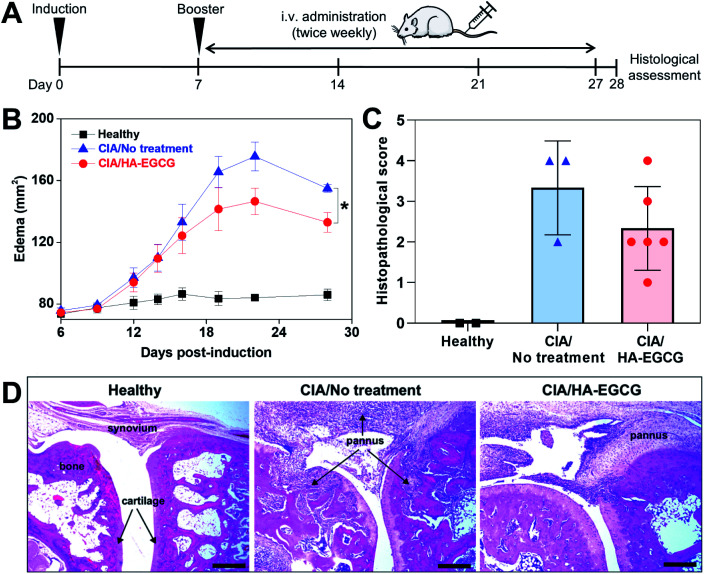
*In vivo* anti-arthritic efficacy. (A) CIA immunization and treatment schedule. (B) Time-course changes in the edema at the hind paws of healthy rats (*n* = 2), CIA rats with no treatment (*n* = 4), and CIA rats treated with HA–EGCG conjugates (*n* = 7, mean ± SEM). *P < 0.05 between two groups. (C) Histopathological scoring for ankle joints of healthy rats (*n* = 2), CIA rats with no treatment (*n* = 3), and CIA rats treated with HA–EGCG conjugates (*n* = 6, mean ± SD). (D) H&E staining images of hind paw ankle joints. Scale bar = 500 μm.

With no precedent for the treatment of HA–EGCG conjugates in CIA rats, a dose of 40 mg kg^−1^ was chosen since the conjugate solution at this dose had a reasonable viscosity for intravenous injection using a 27-gauge needle. We selected intravenous administration because EGCG showed about 3.2-fold higher bioavailability when given intravenously, compared to when given intraperitoneally.^53^ One week after primary immunization, the rats received tail-vein injections of HA–EGCG conjugate solution twice weekly for a total of 7 doses (Fig. 7A). While no paw swelling was detected in healthy rats, swelling of the hind paws appeared in CIA rats on day 12 post-immunization and peaked on day 22 (Fig. 7B). Thereafter, the edema started to subside, possibly due to a spontaneous recovery commonly observed in this RA model. Of note, rats treated with HA–EGCG conjugates showed a significant reduction in the edema compared to the no-treatment group. There was little difference in the body weight change between the two groups throughout the entire study (Fig. S8†), indicating that intravenous administration of HA–EGCG conjugates did not cause any noticeable systemic toxicity.

We also found an overall decrease in the histopathological scores of the rats treated with HA–EGCG conjugates relative to untreated control (Fig. 7C), although a statistical significance was not achieved due to a relatively small sample size. H&E images showed massive pannus formation, cartilage/bone destruction and hyperplasia of the synovial membrane in CIA rats, compared to healthy rats (Fig. 7D), whereas pannus formation and joint destruction were less severe in the rats treated with HA–EGCG conjugates. Collectively, these results demonstrated that HA–EGCG conjugates attenuated the paw swelling and joint destruction in CIA rats, suggesting their potential applicability for RA therapy.

## Conclusions

In this study, we explored the feasibility of HA–EGCG conjugates as an anti-arthritic agent for the treatment of RA. Fluorescence microscopy proved that HA–EGCG conjugates were internalized by FLS through CD44-mediated endocytosis. In addition, these conjugates produced greater amounts of H_2_O_2_ than EGCG in the presence of serum albumin. More importantly, the conjugates effectively suppressed the proliferation and IL-6 production in FLS in a simulated physiological environment. Our preliminary *in vivo* study provided evidence of enhanced accumulation of HA–EGCG conjugates in the inflamed synovium and their beneficial effects in attenuating joint destruction in CIA rats.

## Experimental

### Materials

HA (90 kDa) was kindly donated by JNC Corporation (Japan). EGCG was obtained from DSM Nutritional Products (Switzerland). Dextran (100 kDa), bovine serum albumin (BSA), phosphate-buffered saline (PBS) and incomplete Freund's adjuvant were purchased from Sigma-Aldrich (USA). Fetal bovine serum (FBS) and PicoGreen dsDNA assay kit were purchased from Thermo Fisher (Singapore). Tumor necrosis factor-alpha (TNFα) was purchased from R&D System (USA). Bovine type II collagen was obtained from Chondrex (USA). 2-(*N*-Morpholino)ethanesulfonic acid (MES), *N*-hydroxysuccinimide (NHS), 1-ethyl-3-(3-dimethylaminopropyl)carbodiimide hydrochloride (EDC·HCl), 5-aminofluorescein (AF) and 4,6-diamidino-2-phenylindole (DAPI) were purchased from Sigma-Aldrich (Singapore). 4-(4,6-Dimethoxy-1,3,5-triazin-2-yl)-4-methylmorpholinium chloride (DMTMM) was acquired from Tokyo Chemical Industry Co. Ltd. (Japan). Cyanine7.5 amine dye (Cy7.5) was obtained from Lumiprobe Corporation (USA). Rat anti-human CD44 antibody (clone Hermes-1), isotype control antibody, fluorescein isothiocyanate (FITC)-labeled secondary antibody, Pierce™ quantitative peroxide assay kit and alamarBlue® cell viability assay reagent were purchased from Thermo Fisher Scientific (Singapore). CellTiter-Glo® luminescent cell viability assay reagent was obtained from Promega (Singapore).

### Preparation of HA–EGCG conjugates

HA–EGCG conjugates were synthesized by conjugating ethylamine-bridged EGCG dimers to carboxylic acid groups of HA backbone, according to the previously reported protocol,^29^ and passed through a 0.2 μm syringe filter before lyophilization. The yield was 67% and the degree of substitution (DS) of EGCG dimers in every 100 disaccharide units of HA was 1.8. Stock solution of HA–EGCG conjugates was prepared in deionized H_2_O and stored frozen at −80 °C. HA–EGCG conjugates were thawed to room temperature and diluted with buffer or cell culture medium immediately before use.

### Preparation of HA–EGCG–AF conjugates

HA (500 mg, 1.24 mmol) and AF (208 mg, 0.6 mmol) were dissolved in a mixture containing 16.7 mL of 0.3 M MES buffer (pH 5.2) and 33.3 mL of DMSO. The carboxylic acid groups of HA were activated by DMTMM (18.4 mg, 66 μmol) and the amide coupling reaction was allowed to proceed overnight in the dark with constant stirring. The conjugates were purified by 3 cycles of precipitation using NaCl and EtOH, as described previously.^29^ After the last precipitation, the conjugates were dried for 2 h at 35 °C in a vacuum oven. Yield = 94%. The absorbance of HA–AF conjugates (0.1 mg mL^−1^ in H_2_O) at 488 nm was measured using a UV-Vis spectrophotometer. The DS of AF was 0.8 as determined by comparing the absorbance to a set of AF at known concentrations. Ethylamine-bridged EGCG dimers were then conjugated to HA–AF, according to the previously described protocol.^29^ The emission spectrum of HA–EGCG–AF conjugate was acquired on an Infinite M200 microplate reader (Tecan, Switzerland) with an excitation wavelength at 420 nm.

### Preparation of HA–EGCG–Cy7.5 conjugates

HA (25 mg, 62.2 μmol) was dissolved in 1.1 mL of 0.4 M MEM buffer (pH 5.2), and Cy7.5 (1.5 mg, 1.8 μmol in 0.5 mL of DMF) and DMTMM (2 mg, 7.5 μmol) were added. The reaction was allowed to proceed for 6 h at 45 °C. Next, NHS (4.4 mg, 38.2 μmol), EDC·HCl (8.4 mg, 43.8 μmol) and ethylamine-bridged EGCG dimers (20.5 μmol in 0.24 mL of H_2_O) were added. The reaction mixture was purged with N_2_ for 10 min and incubated for 20 h under N_2_ atmosphere. The conjugates were purified by 3 cycles of precipitation using NaCl and EtOH, and then dialyzed against H_2_O under N_2_ atmosphere overnight. The purified HA–EGCG–Cy7.5 conjugates were passed through a 0.2 μm syringe filter before lyophilization. Yield = 88%. The absorbance of HA–EGCG–Cy7.5 conjugates (0.25 mg mL^−1^ in H_2_O) at 273 nm (*λ*_max_ of EGCG) and 788 nm (*λ*_max_ of Cy7.5) were measured. Based on the absorbance values, the DS of EGCG dimers and Cy7.5 dyes were found to be 1.4 and 0.3, respectively. The emission spectrum of HA–EGCG–Cy7.5 conjugate was acquired on an Infinite M200 microplate reader (Tecan, Switzerland) with an excitation wavelength at 770 nm.

### Cell culture

Human fibroblast-like synoviocytes (FLS) were purchased from Cell Applications, Inc. (USA). FLS were grown in RPMI 1640 medium supplemented with 20 mM HEPES, 10% FBS and 1% penicillin–streptomycin. Cell culture was conducted at 37 °C in a humidified atmosphere containing 5% CO_2_. Media were replaced every 2–3 days and cells were passaged at 80% confluency. FLS below passage 10 were used in this study.

### Flow cytometry

To assess CD44 expression levels, 5 × 10^5^ FLS were incubated with anti-human CD44 antibody or isotype control antibody (2 μg mL^−1^) for 15 min at room temperature. The cells were washed three times with ice-cold PBS containing 10% FBS, and then stained with FITC-labeled secondary antibody for 20 min. Afterwards, the cells were washed again and analyzed by a BD FACSCalibur system (USA). To verify CD44-mediated cellular uptake of HA–EGCG–AF conjugates, FLS were seeded at 1.5 × 10^5^ cells per well in a 12-well plate for 24 h. After removing the spent medium, 500 μL of serum-free medium containing either HA (40 mg mL^−1^; CD44 blocker) or dextran (40 mg mL^−1^; non-CD44 binding polysaccharide) was added to the wells. After 1 h of incubation, 500 μL of HA–EGCG–AF conjugates (0.3 mg mL^−1^) in serum-free medium was added. After incubation for 1 h, the medium was aspirated and the cells were washed with PBS three times to remove residual HA–EGCG–AF conjugates. Then the cells were detached from the wells by trypsin, transferred to an Eppendorf tube and centrifuged at 400 × *g* for 5 min. The supernatant was discarded and the cells were re-suspended in 250 μL of 10% neutral buffered formalin. The cells were fixed for 15 min with gentle mixing by inverting the tube every 5 min. After centrifugation and washing with PBS, the cells were re-suspended in 500 μL of PBS and analyzed by a BD FACSCalibur system.

### Fluorescence microscopy

To investigate cellular uptake of HA–EGCG–AF conjugates, FLS were seeded on 8-well Lab-Tek® II chamber slides at a density of 2 × 10^4^ cells per well and then cultured for 24 h. These cells were treated with HA–EGCG–AF conjugates (0.3 mg mL^−1^) in serum-free medium. At selected time points, the cells were washed with PBS three times and fixed with 4% paraformaldehyde. After re-washing with PBS, the cell nuclei were stained with 4,6-diamidino-2-phenylindole (1.5 μg mL^−1^). The cells were observed under a LSM 510 META confocal laser scanning microscope (Carl Zeiss, Germany).

### Fluorescence quenching study

Quenching of tryptophan fluorescence was used to examine the interactions between BSA and EGCG or HA–EGCG conjugates.^39^ Stock solution of BSA was prepared at 0.4 mg mL^−1^ in 2× PBS. One mL each of BSA stock solution and EGCG or HA–EGCG conjugates (dissolved in deionized H_2_O) were mixed together. The final concentrations were 0.2 mg mL^−1^ for BSA and 0.5 to 8 μM for EGCG or EGCG moieties in HA–EGCG conjugates. The samples were gently mixed by inversion and incubated for 1 min before transferring to a quartz cuvette for fluorescence measurement using a Hitachi F-2500 fluorometer at room temperature. The excitation wavelength was 280 nm and the emission spectra were recorded from 290 to 425 nm. Two independent experiments were performed. Stock solution of BSA in 0.3 M NaCl was used for fluorescence quenching in normal saline.

### H_2_O_2_ quantification

The generation of H_2_O_2_ from EGCG and HA–EGCG conjugates was examined by a Pierce™ quantitative peroxide assay kit. Briefly, EGCG (100 μM), HA–EGCG conjugate or a mixture containing equivalent amounts of EGCG and HA was incubated in 10 mM PBS (pH 7.4) at 37 °C. To investigate the effect of BSA on the H_2_O_2_-producing property of HA–EGCG conjugates in cell culture conditions, EGCG (200 μM) or HA–EGCG conjugate was incubated in RPMI 1640 medium with or without BSA (2% w/v) for 90 min at 37 °C. The amount of H_2_O_2_ in the medium was quantified using Pierce™ quantitative peroxide assay kit according to manufacturer's protocol. Briefly, 20 μL of standards or sample and 200 μL of working reagent were added successively to the wells of a 96-well plate. After incubation for 20 min at room temperature on a shaker, absorbance at 595 nm was measured using a microplate reader (Tecan Infinite M200, Switzerland). The concentration of H_2_O_2_ in the samples was calculated against the standard curve.

### Cell growth assay

FLS were seeded at 2.5 × 10^3^ cells per well in a 96-well plate in 100 μL of complete growth medium. After overnight incubation, the spent medium was replaced with 100 μL of complete growth medium containing TNFα (20 ng mL^−1^), BSA (2% w/v) and EGCG or HA–EGCG conjugates (12.5 to 100 μM). Stock solutions of EGCG and HA–EGCG conjugates were diluted with culture medium immediately before use. After incubation for 3 days, the spent medium was collected and stored at −80 °C for subsequent H_2_O_2_ quantification as described above. The amount of DNA in each well was quantified by Quant-iT PicoGreen assay kit. Briefly, the cells were lysed with 20 μL of Triton X-100 (0.2% v/v in PBS) for 30 min on a shaker. Then 180 μL of PicoGreen working reagent were added to each well. After incubation for 5 min, fluorescence signal was measured using a microplate reader and expressed as percentage of no treatment control. Cell growth assay was also performed using growth media supplemented with catalase (100 units per mL).

### Measurement of IL-6 production

FLS were seeded at 2 × 10^4^ cells per well in 96-well plates and serum-starved for 24 h. These cells were stimulated with TNFα (10 ng mL^−1^) and concurrently treated with EGCG or HA–EGCG conjugates (10–200 μM) in serum-free media with or without 2% BSA. Catalase (100 units per mL) was added into both media at the beginning of cultivation. After 24 h, the amount of IL-6 in the spent medium was determined by sandwich ELISA kit (Abcam, UK).

### Gene expression analysis

To study the effect of HA–EGCG conjugates on IL-6 gene expression, FLS were seeded at 1 × 10^5^ cells per well in a 24-well plate in complete growth medium overnight. After 24 h of serum-starvation, the spent medium was replaced with serum-free RPMI medium containing HA–EGCG conjugates (equivalent to 50 μM of EGCG). After 2 h incubation, the cells were stimulated with TNFα (10 ng mL^−1^). Cells without TNFα stimulation were included as a control. After 24 h of incubation, RNA was isolated from the cells by TRIzol. One microgram of RNA was treated with RQ1 RNase-free DNAse (Promega Corporation). First strand cDNA was synthesized by RevertAid First Strand cDNA Synthesis Kit (Thermo Fisher Scientific) according to manufacturer's instruction. qPCR was performed by SensiFAST™ Probe No-ROX Kit (Bioline) according to manufacturer's protocol using an iQ5 Real-Time PCR System (Bio-Rad). TaqMan® Gene Expression assay was used to amplify and detect IL-6 (assay ID: Hs00985639_m1) expressions. GAPDH (assay ID: Hs02758991_g1) was used as the internal control. The cycle threshold (Ct) values were exported to Microsoft Excel and ΔΔCT was calculated with GAPDH as the reference gene.

### Collagen-induced arthritis (CIA) rat model

All rat experiments were conducted according to the ethical guidelines and protocol approved by the Institutional Animal Care and Use Committee (IACUC) at the Biological Resource Center (BRC) in Biopolis, Singapore. Female Wistar rats (180–230 g) were housed in specific pathogen-free conditions and allowed to acclimatize for five days. Emulsified bovine type II collagen was prepared according to the previous report with some modifications.^54^ Briefly, 10 mg of lyophilized collagen was dissolved at 2 mg mL^−1^ in 0.05 M acetic acid by gentle stirring at 4 °C overnight. To 1.5 mL of collagen solution, an equal volume of incomplete Freund's adjuvant was added dropwise while mixing with a homogenizer at low speed (PRO Scientific Inc., USA). The mixture was kept chilled by submerging in an ice bath and homogenized at medium speed for another 2 min before cooling for 5 min. The homogenization and cooling cycle were repeated twice. The emulsion was kept on ice and used within 2 h of preparation. Rats were anaesthetized by isoflurane and injected with the collagen emulsion (0.2 mL, 200 μg) subcutaneously at the base of tail using a 27-gauge needle. Seven days after primary immunization, a booster injection (0.1 mL, 100 μg) was given at the base of the tail away from the primary injection site. Symptoms of arthritis developed as early as day 12.

### Near-infrared (NIR) fluorescence imaging

Two immunized rats were used for NIR fluorescence imaging study. The first was injected with 1 mL of Cy7.5 dye (47.6 μM in normal saline) and the second with 1 mL of HA–EGCG–Cy7.5 conjugates (7.6 mg mL^−1^ in normal saline). Injections were performed at the tail vein on day 15. The injected amount of Cy7.5 dyes was similar between the two rats. A healthy animal was also injected with HA–EGCG–Cy7.5 conjugates as a comparison. NIR fluorescence images of the ventral and dorsal (shaved) sides of the rats were captured using Xenogen IVIS-200 (excitation wavelength = 745 nm; emission wavelength = 840 nm; exposure time = 2 s; binning = medium) at selected time post-injection. Fluorescence images were analyzed using Living Image® software. The hind paws were designated as the region of interests (ROIs). The total radiant efficiency within ROIs was quantified and the average between the left and right paws was calculated.

### Anti-arthritic efficacy study

Eighteen immunized rats were divided into 3 groups: no treatment (*n* = 4), EGCG treatment (*n* = 7), and HA–EGCG treatment (*n* = 7). Both EGCG and HA–EGCG conjugates were prepared in normal saline. The dose of EGCG (100 mg kg^−1^) and route of administration (intraperitoneal injection) were taken from a previous study.^14^ For HA–EGCG treatment, the rats received tail-vein injection of HA–EGCG conjugates (40 mg kg^−1^) using a 27-gauge needle twice weekly from day 7 to 27 (total 7 doses). Two healthy rats were included as controls. Paw swelling (edema) was measured as previously described.^54^ Briefly, two perpendicular diameters at the ankle and the hind paw were measured using a digital caliper. The cross-sectional areas of the ankle and hind paw were calculated using the formula for the area of an eclipse. Edema score is the sum of the cross-sectional areas of both the left and right ankles and hind paws. The rats were sacrificed on day 28 by CO_2_ asphyxiation. The right ankle joints were collected and processed for histopathological examination.

### Histopathology

Ankle joints were fixed in 10% neutral buffered formalin and decalcified in 5% formic acid. Decalcified tissues were dehydrated, embedded in paraffin, and sectioned for hematoxylin and eosin (H&E) staining. Histopathological score was assessed based on a previously described scale of 0–4, where 0 indicates normal synovial membrane, cartilage and bone, 1 indicates hyperplasia of the synovial membrane, 2 indicates pannus and fibrous tissue formation, 3 indicates moderate destruction of the cartilage and bone, and 4 indicates extensive destruction of the cartilage and bone.^55^

### Statistical analysis

Results were expressed as the mean ± standard deviation (SD) or standard error of the mean (SEM). The difference between two groups was assessed using Student's *t*-test in Microsoft Excel, whereas the difference among multiple groups was assessed using one-way ANOVA in OriginPro 2016.

## Author contributions

Fan Lee: conceptualization, investigation, formal analysis, writing – original draft. Ki Hyun Bae: formal analysis, visualization, writing – review & editing. Shengyong Ng: investigation, formal analysis, writing – review & editing. Atsushi Yamashita: investigation, formal analysis. Motoichi Kurisawa: conceptualization, funding acquisition, project administration, resources, supervision, writing – review & editing.

## Conflicts of interest

There are no conflicts to declare.

## Supplementary Material

RA-011-D1RA01491A-s001
