# Supplementary material for: Hyaluronic acid–green tea catechin conjugates as a potential therapeutic agent for rheumatoid arthritis
Source: RSC Adv. 2021 Apr 19;11(24):14285–94. doi: 10.1039/d1ra01491a (PMC8697692; doi:10.1039/d1ra01491a)
Supplement: RA-011-D1RA01491A-s001 [file RA-011-D1RA01491A-s001.pdf]

Electronic Supplementary Material (ESI) for RSC Advances.
This journal is © The Royal Society of Chemistry 2021

Supplementary Materials for

Hyaluronic Acid-Green Tea Catechin Conjugates as a Potential Therapeutic Agent for Rheumatoid Arthritis

Fan Lee, Ki Hyun Bae, Shengyong Ng, Atsushi Yamashita, Motoichi Kurisawa*

Institute of Bioengineering and Bioimaging, 31 Biopolis Way, The Nanos, Singapore 138669, Singapore

*Tel.: +65-6824-7139. Fax: +65-6478-9083. E-mail: mkurisawa@ibn.a-star.edu.sg

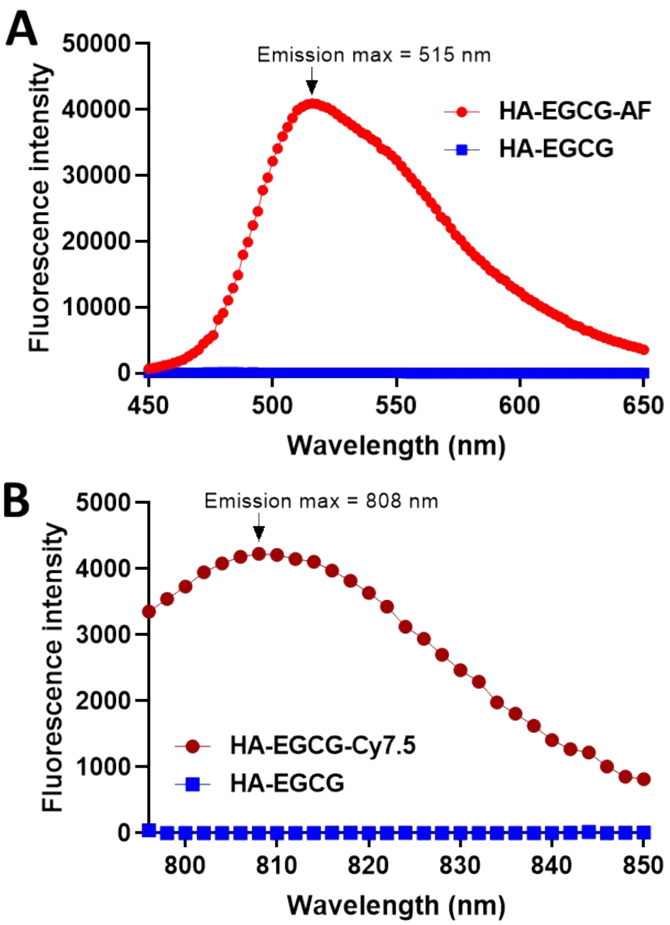

Fig. S1. Fluorescence emission spectrum of (A) HA-EGCG-AF and (B) HA-EGCG-Cy7.5 conjugates in deionized water (1 mg mL^{-1}). HA-EGCG conjugates without fluorescence labeling were also tested for comparison. The arrows indicate the wavelength maximum of fluorescence emission.

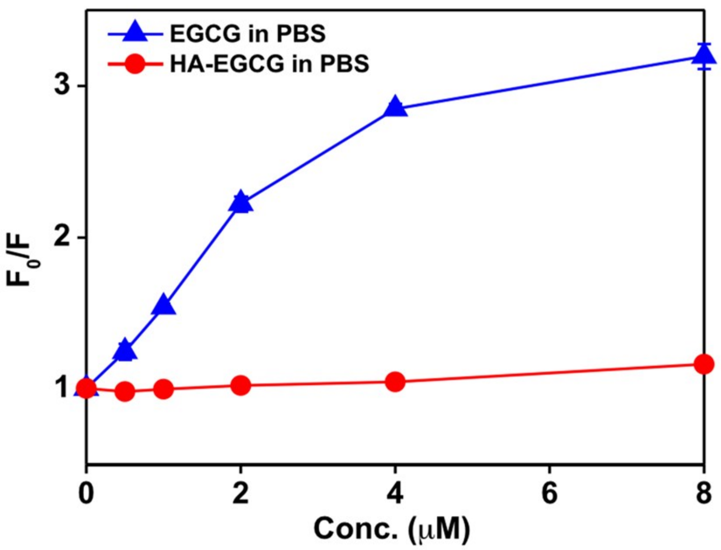

Fig. S2. Stern−Volmer plots for the quenching of BSA fluorescence upon addition of increasing amounts of EGCG or HA-EGCG conjugates. F_0 and F are the fluorescence intensities of BSA (λ_{ex} = 280 nm, λ_{em} = 341 nm) in the absence and presence of the quencher (EGCG or HA-EGCG conjugates), respectively.

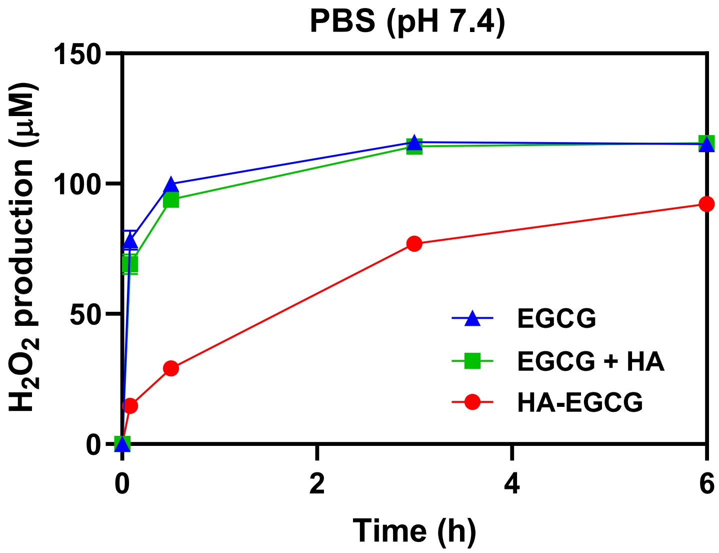

Fig. S3. Time course of H_2O_2 production by EGCG and HA-EGCG conjugates in 10 mM PBS (pH 7.4) at 37 ºC (n = 2, mean ± SD). The concentration of EGCG was fixed at 100 μM. A physical mixture containing equivalent amounts of EGCG and HA was also tested for comparison.

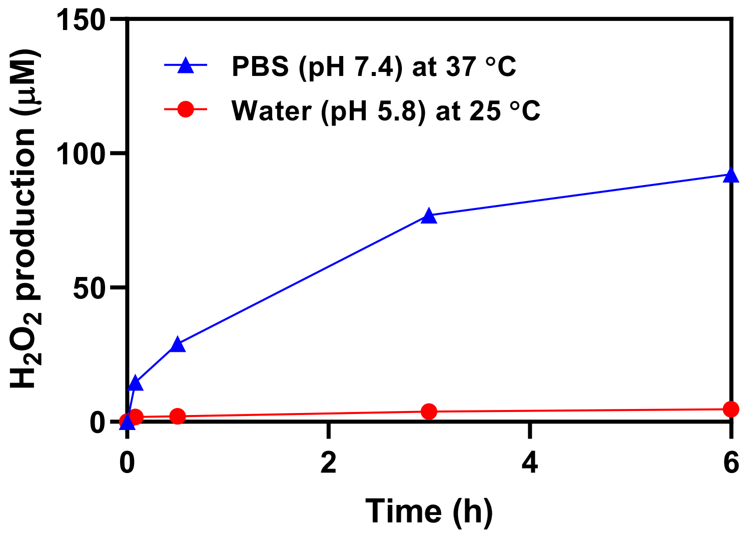

Fig. S4. Time course of H_2O_2 production by HA-EGCG conjugates in 10 mM PBS (pH 7.4) at 37 °C or deionized water (pH 5.8) at 25 °C (n = 2, mean ± SD). The concentration of EGCG was fixed at 100 μM.

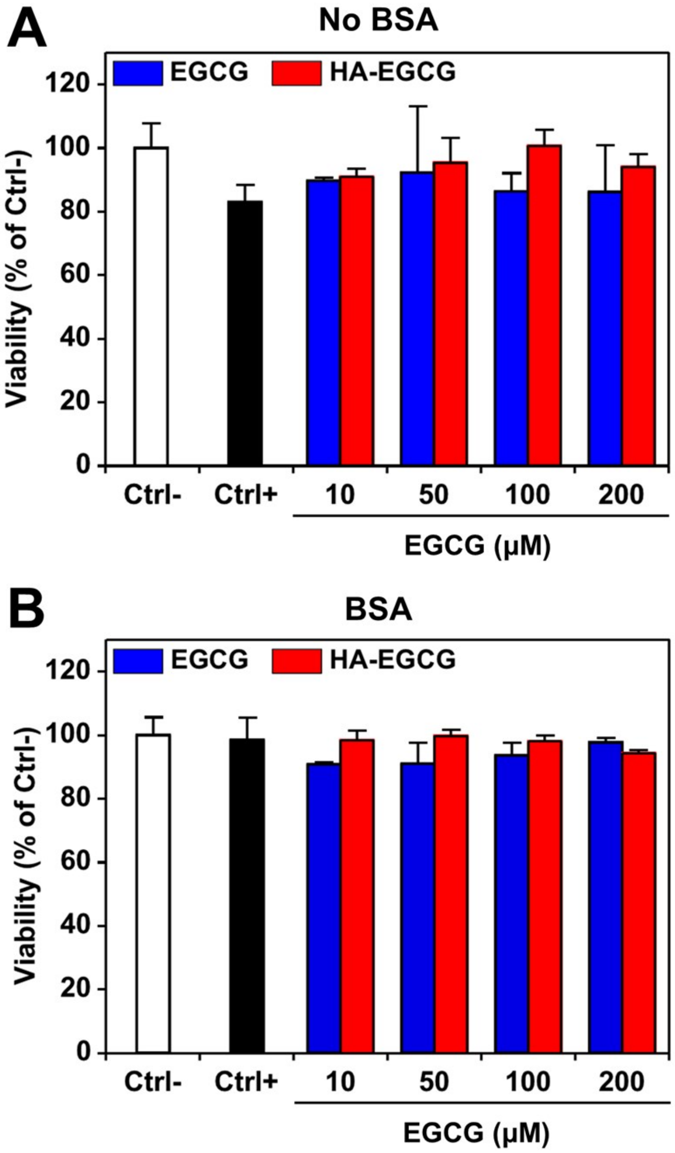

Fig. S5. Viability of FLS after 24 h treatment of TNFα-stimulated FLS with EGCG or HA-EGCG conjugates in (A) RPMI medium and (B) BSA-supplemented RPMI medium (n = 2, mean ± SD). Catalase (100 units mL^{-1}) was added into both media at the beginning of cultivation. Ctrl-: negative control (unstimulated FLS), Ctrl+: positive control (TNFα-stimulated FLS).

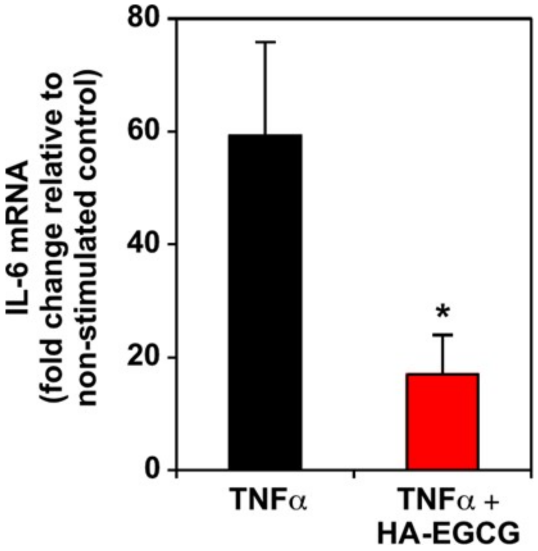

Fig. S6. IL-6 mRNA expression levels of TNFα-stimulated FLS treated with or without HA-EGCG conjugates (equivalent to 50 μM of EGCG). The data are presented as mean ± SD (n = 3). *P < 0.05 versus TNFα-stimulated FLS.

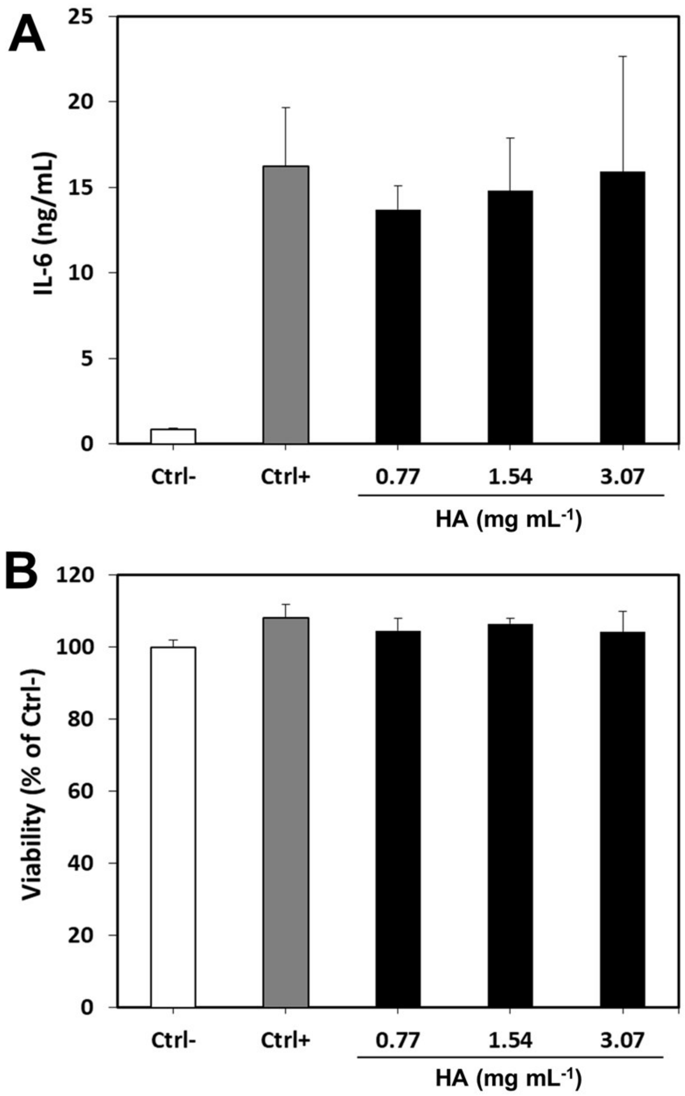

Fig. S7. Effect of HA alone on (A) IL-6 production and (B) Viability of FLS (n = 2, mean ± SD). FLS were treated with varying concentrations of HA for 24 h in RPMI medium containing TNFα (10 ng mL^{-1}) and catalase (100 units mL^{-1}). The concentrations of HA (0.77, 1.54 and 3.07 mg mL^{-1}) correspond to those of HA-EGCG conjugates containing 50, 100 and 200 μM of EGCG, respectively.

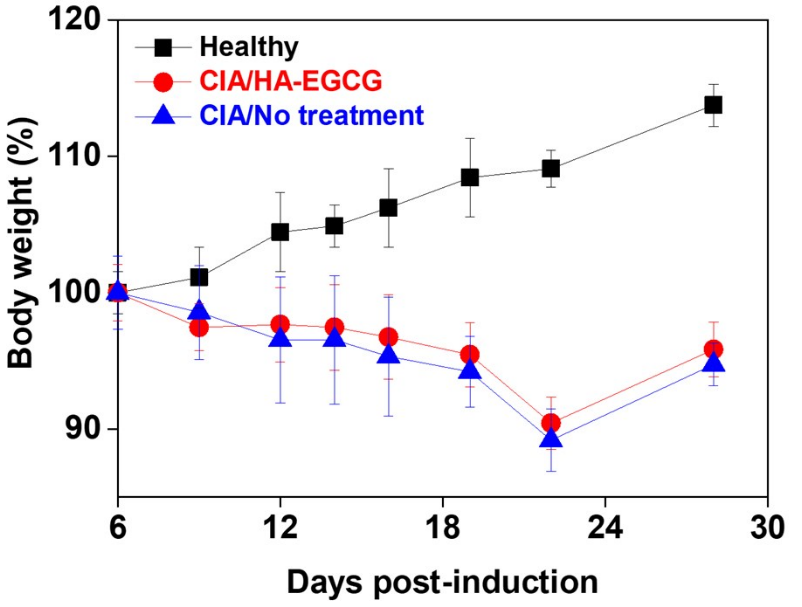

Fig. S8. Time course of relative body weight changes in healthy rats (n = 2), CIA rats with no treatment (n = 4), and CIA rats treated with HA-EGCG conjugates (n = 7, mean ± SEM).
